# High-Flow Nasal Cannula: A Promising Oxygen Therapy for Patients with Severe Bronchial Asthma Complicated with Respiratory Failure

**DOI:** 10.1155/2020/2301712

**Published:** 2020-02-20

**Authors:** Wanru Geng, Wuliji Batu, Shuhong You, Zhaohui Tong, Hangyong He

**Affiliations:** ^1^Department of Respiratory and Critical Care Medicine, Beijing Institute of Respiratory Medicine, Beijing Chao-Yang Hospital, Capital Medical University, Beijing 100000, China; ^2^Department of Respiratory Medicine, The Hospital Affiliated to the Inner Mongolia University for Nationalities, Tongliao 028000, China

## Abstract

Severe bronchial asthma complicated with respiratory failure, a common critical illness in respiratory medicine, may be life-threatening. High-flow nasal cannula (HFNC) is a novel oxygen therapy technique developed in recent years. HFNC was applied in this study for treating adult patients with severe bronchial asthma complicated with respiratory failure. Its efficacy was analyzed comparatively to conventional oxygen therapy (COT). HFNC and COT were randomly performed based on conventional treatment. The HFNC group was similar to COT-treated patients in terms of response rate, with no significant difference in efficacy between the two groups. In patients with bronchial asthma, effectively increased PO_2_ and reduced PCO_2_ were observed after treatment in both groups. However, HFNC was more efficient than COT in elevating PO_2_ in patients with severe bronchial asthma complicated with respiratory failure, while no statistically significant difference in PCO_2_ reduction was found between the two groups. Heart rate (HR) and respiratory rate (RR) between the two groups on admission (0 h) and at 2, 8, 24, and 48 h after admission were compared. Both indicators significantly decreased with time. No significant differences in HR and RR were found between the groups at 0, 2, and 8 h after admission. However, these indicators were significantly lower in the HFNC group compared with the COT group at 24 and 48 h after admission. HFNC could significantly elevate PO_2_ and reduce HR and RR. Thus, it is a promising option for patients with severe bronchial asthma complicated with respiratory failure.

## 1. Introduction

Bronchial asthma is a chronic inflammatory disease of the airways characterized by cough, paroxysmal wheezing, and chest tightness. Approximately 300 million individuals are affected by asthma worldwide [1]. In China, a recent national survey showed an overall incidence of 4.2%, with about 457 million asthma cases among adults [2]. Severe bronchial asthma combined with respiratory failure is clinically diagnosed by persistent dyspnea and airway obstruction, or even more severe symptoms, which may be life-threatening. Hence, active rescue and treatment at the emergency or respiratory department are often necessary [3]. In addition to conventional symptomatic treatments, such as repeated inhalation of SABA, oral glucocorticoids, and other drugs for relieving asthma, oxygen therapy is an essential adjuvant strategy [3, 4]. Currently, conventional oxygen therapies, such as the use of nasal cannula, venturi mask, and non-rebreathing bag reservoir face mask, are mainly used in clinical settings, but sufficient respiratory support cannot be ensured, thereby increasing the likelihood of invasive mechanical ventilation [5].

HFNC is a novel oxygen therapy developed in recent years, in which oxygen at a certain concentration mixed with high-flow gas is directly delivered to patients through a nonsealed nasal cannula [6, 7]. This oxygen therapy has been assessed by many studies and can be applied to patients with acute hypoxemic respiratory failure, status-post surgery, and respiratory failure without tracheal intubation, immunosuppression, and cardiac insufficiency, thus effectively improving oxygenation [8–15]. However, few studies have confirmed the efficacy of HFNC in treating bronchial asthma, focusing mainly on pediatric and neonatal patients [16, 17]. However, HFNC in adult patients with severe bronchial asthma complicated with respiratory failure has not been reported. Thus, this study was designed to analyze the efficacy of HFNC in adult patients with severe bronchial asthma.

## 2. Materials and Methods

### 2.1. Study Design

This study was performed in the Affiliated Hospital of Inner Mongolia University for Nationalities (Inner Mongolia Autonomous Region, China). It included adult patients with acute severe bronchial asthma complicated with respiratory failure admitted to the above hospital between June 2017 and January 2019. It was a single-center, open-label, randomized controlled trial designed to analyze the efficacy and safety of HFNC versus COT in improving oxygenation. The present study was approved by the Ethics Committee of the Affiliated Hospital of Inner Mongolia University for Nationalities, and patients who agreed to participate were required to sign the informed consent form.

### 2.2. Participants

From June 2017 to January 2019, adult patients with a primary diagnosis of acute severe bronchial asthma complicated with respiratory failure, admitted to the emergency, pulmonary medicine, and intensive care medicine departments of the hospital, were enrolled in this study.

A total of 3862 patients aged 18–75 years initially diagnosed with bronchial asthma were enrolled. Diagnosis of adult severe bronchial asthma with respiratory failure was performed independently by two attending physicians based on the following inclusion criteria. In case of discrepant diagnosis, the patient was excluded.

Inclusion criteria were as follows: (1) acute severe bronchial asthma confirmed according to the Global Initiative for Asthma (GINA) diagnostic criteria; (2) PO_2_ < 60 mmHg, with or without PCO_2_ ≥ 45 mmHg, under room air according to blood gas analysis on admission.

Exclusion criteria were as follows: (1) immediate requirement of tracheal intubation; (2) myocardial infarction (cardiothoracic pain with electrocardiogram changes or increased levels of myocardial enzymes); (3) altered consciousness; (4) hemodynamic involvement (noninvasive blood pressure < 90/60 mmHg); (5) pregnancy in women; (6) respiratory rate (RR) > 45 breaths/min; (7) blood pH < 7.30; (8) untreated pneumothorax; (9) end-stage renal disease [estimated glomerular filtration rate (eGFR) < 15 mL/min per 1.73 m^2^ or current dialysis]; (10) contraindications for positive–airway pressure devices; (11) pneumonia.

Of the 53 eligible patients included in this study, 17 were excluded, including 4 complicated with myocardial infarction, 2 with pneumothorax, 1 with pregnancy, 2 with pneumonia, 2 with decreased blood pressure, and 6 with refusal to provide a signed informed consent. Therefore, 36 patients were finally enrolled. The SAS software PROC PLAN was used to randomize the blocks (block length of 4), and the 36 patients were randomly assigned to the HFNC and COT groups. Among them, 20 were randomized to the COT group (2–6 L/min for oxygen inhalation with a nasal cannula, and oxygen inhalation with a venturi or storage mask); 16 were randomly assigned to the HFNC group according to the corresponding treatments. Subsequently, all patients were evaluated and grouped according to diagnosis by more than two attending physicians. Informed consent was obtained, and standard treatments (a short-acting *β*2 agonist, inhaled or intravenous corticosteroids, and antibiotics, if necessary) were ensured.

### 2.3. Interventions

For basic treatment, the therapeutic regimens were codetermined by two attending physicians based on the GINA criteria, including aerosol inhalation drugs, salbutamol inhalation, use of systemic glucocorticoids and magnesium sulfate, and adjustment of drug regimen with condition change.

In the HFNC group, oxygen therapy was performed with an AIRVO-2 respiratory humidified therapeutic apparatus (Fisher & Paykel Healthcare Company). Therapeutic regimens were codetermined by two attending physicians, and ventilator therapists with specialized training for more than 3 months were responsible for adjusting the parameters of the high-flow apparatus. Parameter settings for the HFNC apparatus were as follows: (1) initial gas flow of 30–40 L/min; (2) FiO_2_ titration to maintain pulse oxygen saturation (SpO_2_) at 92%–96%; (3) blood gas analysis for dynamic adjustment (in case of no proper oxygenation, the inspiratory flow could be increased gradually and FiO_2_ could be increased up to 100%); (4) temperature range of 31–37°C appropriately adjusted according to patient comfort and tolerance as well as sputum viscosity. HFNC weaning criteria were as follows: (1) HFNC parameters gradually decreased after gradual control of asthma. If the following criteria were met, HFNC weaning was considered: inspiratory flow < 20 L/min and FiO_2_ < 30%. The flow of HFNC was initially set at 30–40 L/min, and inspiratory flow rate could be increased to 45–60 L/min to achieve the maximum flow tolerated by the patients.

In the COT group, conventional oxygen inhalation methods, including nasal cannula, venturi mask, and storage balloon mask, were used. According to the patient's condition, the appropriate oxygen inhalation mode and oxygen flow were determined. The appropriate oxygen therapy was determined independently by the two attending physicians. Ventilator therapists were responsible for all procedures involved in oxygen inhalation to maintain SpO_2_ within a range of 92%–96%, and blood gas analysis was combined for dynamic adjustment.

### 2.4. Outcome Measures

#### 2.4.1. Primary Outcomes

Primary outcomes were defined as the clinical efficacies of different oxygenation methods based on previous studies [18, 19]. They were classified into three categories: (1) improvement (controlled acute symptoms, unremarkable laboratory indexes, and auscultation of lungs showing wheezing disappearance); (2) effectiveness (alleviated acute symptoms, RR reduction by 20%, heart rate (HR) reduction by 20%, FiO_2_ < 0.5, and auscultation of lungs showing reduced wheezing); and (3) ineffectiveness (patients not fulfilling the above criteria for improvement or effectiveness, emergence of new symptoms or signs, or further condition deterioration, requiring noninvasive positive pressure ventilation or intubation). Efficacy was determined by two attending physicians independently. The response rate was derived as (number of cases with improvement + number of cases with effectiveness)/total number of study patients × 100%.

#### 2.4.2. Secondary Outcomes

After admission, RR and HR were measured using a multiparameter vital sign monitor (GE) and recorded at the beginning of oxygen therapy (0 h) and 2, 8, 24, and 48 h after oxygen therapy. In addition, a blood gas analyzer (Roche) was used to detect blood gases in patients under no oxygen inhalation. PaO_2_ and PaCO_2_ obtained on admission were used as baseline levels (0 h), and these parameters were also recorded during weaning or discontinuing oxygen therapy. Blood gas analysis was performed, in case of condition change.

### 2.5. Data Collection

On admission, patient baseline characteristics, including age, weight, sex, comorbidities, and asthma history, were recorded. In addition, hospital stay, duration of HFNC, and the time of oxygen therapy were assessed, as well as safety.

### 2.6. Statistical Analysis

Based on the controlled trial design for two intervention groups, with a binominal primary endpoint [20], the sample size was calculated. *α* = 0.05 and *β* = 0.20 were set. Based on response rates for control patients and HFNC cases reported in a previous study [19, 21] and unpublished data obtained in our center describing the failure rate of conventional oxygenation therapy, we estimated at least a total of 34 subjects with an expected reactive rate of 95% in the HFNC group and 55% (95% ^*∗*^[1−0.4] = 57%; a 40% reduction) in the control group (confidence level [1−*α*] = 95% and power level[1−*β*] = 80%).

Measurement data are $\bar{x}\pm s$. Changes in variables with time were assessed by repeated measures one-way analysis of variance (ANOVA), with differences between variables detected by Fisher's test. Normally distributed variables were compared by the *t*-test, and those with skewed distribution by the rank-sum test. Percentages were compared by the *χ*^2^ test. Two-sided *P* < 0.05 was considered statistically significant. The SPSS software (SPSS, USA) was used for data analysis.

## 3. Results

### 3.1. Baseline Characteristics

**Figure 1:**
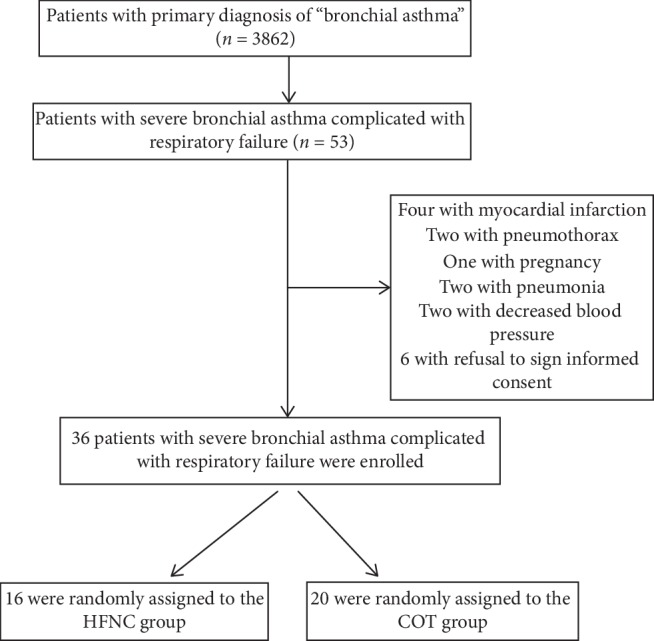
Flowchart of the patient selection process.

In this study, a total of 3,862 patients were diagnosed with bronchial asthma. Of these, 53 met the above inclusion criteria. According to exclusion criteria, 36 were eventually enrolled, with 16 in the HFNC group and 20 in the COT group. Oxygen therapy was administered with a nasal cannula, venturi mask, and storage mask in 13, 4, and 3 patients, respectively (Figure 1). No statistically significant differences in baseline demographic characteristics and asthma severity were found between the two groups (*P* > 0.05) (Table 1).

### 3.2. Therapeutic Outcomes

The HFNC group was similar to COT treated patients in terms of response rate (93.75% vs 95%, *P* = 0.87), with no significant difference in efficacy between the two groups (Table 2).

In patients with bronchial asthma for both groups, PO_2_ was effectively elevated (54.65 ± 6.86 vs 94.73 ± 4.43, 52.68 ± 8.42 vs 86.98 ± 6.42, *P* < 0.05) after the treatment, while PCO_2_ was reduced (51.20 ± 8.75 vs 40.22 ± 4.3, 48.71 ± 3.32 vs 39.87 ± 4.35, *P* < 0.05). However, the between-group comparison showed that the HFNC group was more efficient compared with the COT group in elevating PO_2_ in patients with severe bronchial asthma complicated with respiratory failure. No statistically significant difference in the effect on PCO_2_ was found between the two groups (*P* > 0.05) (Table 3).

**Figure 2:**
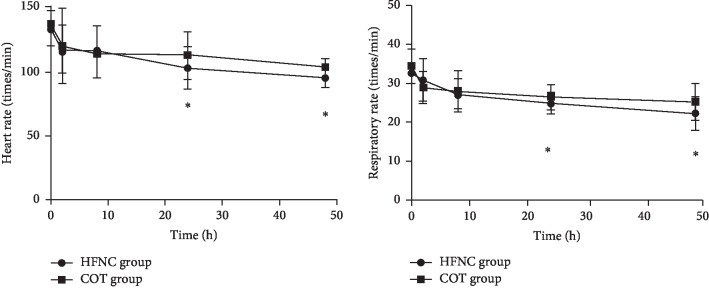
Respiratory rate and heart rate of patients follow a downward trend with time. The statistical data indicated no significant differences between the two treatments at 0, 2, and 8 h but heart rate and respiratory rate were lower in the HFNC group compared with the COT group from 24 h to 48 h (^*∗*^*P* < 0.05).

HR and RR in both groups on admission (0 h) and at 2, 8, 24, and 48 h after admission were assessed. The results suggested that these indicators in both groups significantly decreased with time. No significant differences in HR and RR were observed between the two groups at 0, 2, and 8 h after admission (*P* > 0.05). However, both indicators were significantly lower in the HFNC group compared with the COT group at 24 and 48 h after admission (^*∗*^*P* < 0.05) (Figure 2).

Finally, hospital stay, HFNC duration, and oxygen therapy time in both groups were assessed. The results showed no significant differences in tracheal intubation rate, hospital stay, and duration of oxygen therapy between the two groups (*P* > 0.05) (Table 4).

## 4. Discussion

The present study showed that HFNC had the same clinical efficacy as COT in patients with severe bronchial asthma complicated with respiratory failure, with no significant differences in tracheal intubation rate and hospital stay between the two groups. Although adult patients with bronchial asthma are rarely studied, the current observations were basically consistent with previous reports on children with moderate-to-severe asthma treated with HFNC [17, 22]. Ballestero et al. used PS scores in children with moderate-to-severe bronchial asthma treated with HFNC to grade dyspnea symptoms. They found significantly better PS scores in the HFNC group compared with the COT group during the first 2 h (16/30 vs 9/32, *P*=0.01), but no significant differences in hospital stay, further treatments, and overall efficacy were noted between the two groups [22].

We also demonstrated that, compared with COT, HFNC effectively elevated PO_2_ in patients with severe bronchial asthma complicated with respiratory failure. Although related studies on HFNC in treating adult patients with asthma are rare, these findings corroborated previous studies evaluating type I respiratory failure [23, 24]. Many studies on severe pneumonia, ARDS, status-post surgery, and weaning of mechanical ventilation showed that HFNC elevates PO_2_ in patients with type I respiratory failure and mild type 2 respiratory failure [8–11, 25]. The pathophysiological mechanisms of severe bronchial asthma complicated with respiratory failure are as follows: (1) airway spasm further causes ventilatory dysfunction, leading to respiratory failure; (2) hyperventilation and excessive sweating leading to water loss cause difficulty in expectorating and further result in ventilatory dysfunction and its exacerbation; and (3) infection causes bronchial mucosal edema, increases secretions, aggravates obstruction, and ultimately leads to respiratory failure [1, 23].

In the present study, no difference in PCO_2_ levels was found between the two groups, corroborating previous reports assessing hypercapnic patients [26, 27]. Studies have shown that, in COPD patients with elevated CO_2_, HFNC increases tidal volume, reduces respiratory rate, and decreases CO_2_ [28, 29]. Other reports even showed that CO_2_ is increased after HFNC therapy [30]. These discrepancies may be due to the small sample sizes and differences in patient characteristics. However, we believe that the main reason is that HFNC effectiveness is flow- and leakage-dependent. That we found no CO_2_ reduction in asthmatic patients may be explained by findings reported by Braunlich et al. in COPD patients [31]. The latter study demonstrated that effective PCO_2_ reduction by HFNC therapy does not correlate with mean airway pressure increase, but with elevated leakage and airflow. This indicates airway washout and the reduction of functional dead space as important mechanisms of HFNC therapy. Although we used a relatively high flow rate, leakage-inducing techniques were not applied to the patients, which may explain the relatively low efficiency in CO_2_ reduction in this study.

Based on conventional treatments, such as intravenous and nebulized inhaled glucocorticoids, patients with severe bronchial asthma complicated with respiratory failure must undergo oxygen therapy to maintain oxygenation. HFNC, as a novel oxygen therapy method, provides high-flow gas at the rate of 8–80 L/min through the air–oxygen mixing device, and the oxygen concentration of these gases can be set to 21%–100% according to the patient's needs, thus theoretically meeting the needs for improved oxygenation [25]. In addition, oxygen therapy using high-flow gas also ensures a high inspiratory flow rate in patients with bronchial asthma as well as stable oxygen delivery [6]. The efficacy of the COT method may be low due to insufficient oxygen content in the air; alternatively, the flow rate may not meet the needs of patients' conditions, and hence the effect of oxygen supply cannot be ensured [32].

Similar to previous findings about acute dyspnea [32], HFNC could effectively reduce RR and HR in this study, providing heated and humidified gas. The heated and humidified effect of HFNC on the inhaled gas accounts for its use in the effective treatment of adult patients with severe bronchial asthma complicated with respiratory failure. Normal adults lose about 300–500 mL of water per day through the respiratory tract. In case of severe bronchial asthma, this water loss suddenly increases, easily leading to sputum formation, aggravating the condition [33]. HFNC provides humidified (relative humidity close to 100%) and heated (31–37°C) gas to the patient through its active humidifier and guidewire with heat shrink tubing. Williams and collaborators conducted a comprehensive analysis of more than 200 studies and proposed that this heated and humidified gas maintains the integrity of the airway epithelium, helps dilute the sputum and promotes its removal by the ciliated epithelium, and reduces airway inflammation [34].

Other studies suggested that inhalation of heated and humidified gases is particularly more important in patients with bronchial asthma compared with those suffering from other respiratory diseases [35]. Inhaling dry and cold gases is a stimulating factor in bronchial asthma. Compared with HFNC, conventional nasal catheter oxygen therapy can only deliver unheated dry and cold gases, which causes airway inflammation, increases airway resistance, damages mucociliary function, and hinders the clearance of secretions, thus further aggravating the condition [36].

Moreover, HFNC can meet the requirements of a high flow rate for inhaled gases in patients with respiratory failure, thereby reducing the work of breathing and significantly lessening both HR and RR. In adults with severe bronchial asthma complicated with respiratory failure, the maximum inspiratory flow rate can be 30–60 L/min according to the asthma grading standard [37]. HFNC can provide high-flow gas at a rate of 8–80 L/min, meeting the respiratory requirements of patients with asthma. Meanwhile, COT does not provide sufficient respiratory support for patients with severe bronchial asthma; patients need extra effort to breathe and increase the work of breathing to maintain oxygenation. In 2015, Pham et al. reported significantly decreased electric activity of the diaphragm upon HFNC administration, further confirming that HFNC could effectively reduce the work of breathing [38].

The limitations of this study should be mentioned. First, there was no follow-up evaluation, which could help assess the recurrence rate of asthma, the number of days of acute attack in a year, the 90-day mortality rate, and the time to discharge. This could better illustrate the therapeutic value of the high-flow oxygen inhalation technology. In addition, the sample size was relatively small, and no multicenter, large-scale prospective randomized controlled trials have provided evidence on the clinical guidance for efficiently treating patients with severe bronchial asthma complicated with respiratory failure. Thus, multicenter clinical studies should be conducted in the future to address these shortcomings. Finally, in the present study, no difference in PCO_2_ levels was found between the two groups, likely because leakage-inducing techniques were not applied to the patients, which should be considered in the future studies.

## 5. Conclusions

The current study showed that HFNC is generally consistent with COT in terms of overall clinical response rate in patients with severe bronchial asthma and respiratory failure but could significantly elevate PO_2_ and reduce HR and RR. Thus, HFNC is a promising option for such patients.

## Figures and Tables

**Table 1:** Baseline characteristics of patients in the HFNC and COT groups.

Variables	HFNC group (*n* = 16)	COT group (*n* = 20)	*P* value
Female, sex, *n* (%)	10 (62.5%)	12 (60%)	0.515
Age (year)	43.3 ± 10.6	37.5 ± 8.4	0.08
Median ± SD			
Medical history of asthma (year)	6.38 ± 1.28	5.95 ± 1.36	0.33
Venous blood gas before the trial			
pH	7.30 ± 0.12	7.25 ± 0.58	0.35
PCO_2_	51.20 ± 8.75	48.71 ± 3.32	0.24
PO_2_	54.45 ± 6.86	52.68 ± 8.42	0.50
HR	134.0 ± 11.32	138.32 ± 17.54	0.40
RR	32.71 ± 2.40	34.54 ± 4.34	0.11
Treatments before the trial			
Oxygen in hospital, *n* (%)	16 (100%)	19 (95%)	0.55
Corticosteroids	16 (100%)	19 (95%)	0.55

**Table 2:** Therapeutic effects of HFNC and COT in patients with severe asthma exacerbation combined with respiratory failure.

	Total number	Number of patients with effectiveness	Number of patients with improvement	Number of patients with ineffectiveness	Response rate	*P*
HFNC group	16	8	7	1	15 (93.75%)	Chi-square = 0.026
COT group	20	11	8	1	19 (95%)	0.87

Comparison between the two groups, ^*∗*^*P* < 0.05; between-group comparison, ^#^*P* < 0.05.

**Table 3:** Effects of HFNC and COT on PO_2_ and PCO_2_ in severe asthma exacerbation patients combined with respiratory failure.

Group	Oxygen therapy	PO_2_	PCO_2_
HFNC group	Pretreatment	54.45 ± 6.86	51.20 ± 8.75
	Posttreatment	94.73 ± 4.43^*∗*^	40.22 ± 4.37^*∗*^

COT group	Pretreatment	52.68 ± 8.42	48.71 ± 3.32
	Posttreatment	86.98 ± 6.42^*∗*#^	39.87 ± 4.35^*∗*^

**Table 4:** Medical indicators in the HFNC and COT groups.

Variable	HFNC group (*n* = 16)	COT group (*n* = 20)	*P* value
Intubation (person)	1	1	0.69
Hospitalization days (d)	6.54 ± 1.85	7.02 ± 2.32	0.24
Oxygen days (d)	5.76 ± 1.38	6.43 ± 1.82	0.10

## Data Availability

The data used to support the findings of this study are available from the corresponding author upon request.

## Abbreviations

GINA: Global initiative for asthma
HFNC: High-flow nasal cannula
COT: Conventional oxygen therapy
HR: Heart rate
RR: Respiratory rate
GINA: Global initiative for asthma.
